# Experience of Laparoscopic Exploration and Gonadectomy in lntersex Children

**DOI:** 10.1155/DTE.4.127

**Published:** 1998

**Authors:** Ken Morita, Katsuya Nonomura, Kaname Ameda, Hidehiro Kakizaki, Toshiki Koyama, Tetsufumi Yamashita, Masashi Murakumo, Tomohiko Koyanagi

**Affiliations:** Department of Urology Hokkaido University School of Medicine N15, W7, Kita-Ku Sapporo 060 Japan

## Abstract

The use of laparoscopic technique to diagnose and treat intersex children is gradually introduced in clinical urology. From 1985 to 1996, abdominal exploration and gonadectomy were performed in 11 intersex children together with urogenital endoscopy and genitoplastic surgery in our institutes. Their median age was 6.0 (range 0–15) years old and initial gender sex was female in 8 and male in 3. The initial 4 cases (group 1) underwent open abdominal exploration together with gonadectomy, while the latter 7 cases (group 2) underwent laparoscopic exploration simultaneously with 3 laparoscopic gonadectomy and 1 open one via a inguinal incision. Their final diagnoses were male pseudohermaphroditism in 4 cases, mixed gonadal dysgenesis in 3, true hermaphroditism in 2, XX gonadal dysgenesis in 1, and XY gonadal dysgenesis in 1. Consequently, 2 of initial male were reared as a female. Operation time, use of analgesics, postoperative hospital stay and postoperative complications were not significantly different between the two groups, however, postoperative abdominal wound appearance was more acceptable in group 2. The most significant advantage of laparoscopic surgery in intersex children is cosmetic appearance especially when social gender is determined as female irrespective types of intersexuality.


*Diagnostic and Therapeutic Endoscopy*, Vol. 4, pp. 127–133
Reprints available directly from the publisher
Photocopying permitted by license only

© 1998 OPA (Overseas Publishers Association)
Amsterdam B.V. Published under license
under the Harwood Academic Publishers imprint,
part of The Gordon and Breach Publishing Group.
Printed in Singapore.

### Experience of Laparoscopic Exploration and Gonadectomy in Intersex Children

KEN MORITA *, KATSUYA NONOMURA, KANAME AMEDA, HIDEHIRO KAKIZAKI, TOSHIKI KOYAMA, TETSUFUMI YAMASHITA, MASASHI MURAKUMO and TOMOHIKO KOYANAGI

*Department of Urology, Hokkaido University School of Medicine, N15, W7, Kita-Ku, Sapporo, 060, Japan*

*(Received 28 April 1997; In final form 1 July 1997)*

**The use of laparoscopic technique to diagnose and treat intersex children is gradually introduced in clinical urology. From 1985 to 1996, abdominal exploration and gonadectomy were performed in 11 intersex children together with urogenital endoscopy and genitoplastic surgery in our institutes. Their median age was 6.0 (range 0–15) years old and initial gender sex was female in 8 and male in 3. The initial 4 cases (group 1) underwent open abdominal exploration together with gonadectomy, while the latter 7 cases (group 2) underwent laparoscopic exploration simultaneously with 3 laparoscopic gonadectomy and 1 open one via a inguinal incision. Their final diagnoses were male pseudohermaphroditism in 4 cases, mixed gonadal dysgenesis in 3, true hermaphroditism in 2, XX gonadal dysgenesis in 1, and XY gonadal dysgenesis in 1. Consequently, 2 of initial male were reared as a female. Operation time, use of analgesics, postoperative hospital stay and postoperative complications were not significantly different between the two groups, however, postoperative abdominal wound appearance was more acceptable in group 2. The most significant advantage of laparoscopic surgery in intersex children is cosmetic appearance especially when social gender is determined as female irrespective types of intersexuality.**

*Keywords:* Laparoscopic surgery, Minimally invasive, Intersex, Children

### INTRODUCTION

Recently laparoscopic surgery has been widely used in the field of urology. As a minimally invasive therapeutic modality, laparoscopic surgery has been applied for adrenalectomy, nephrectomy, pelvic lymphnode dissection and others. On the other hand, laparoscopy in nonpalpable testis or intersex has been used as a diagnostic tool for defining internal gonadal and ductal structures as well as their vascular supply. Observational laparoscopy can determine the subsequent appropriate

* Corresponding author. Tel.: +81-11-716-1161 Ext. 5949 (daytime), +81-11-747-0850 (night). Fax: +81-11-736-8052.

management for gonad, which could be performed with open surgical as well as laparoscopic procedures.

Until 1990, children with ambiguous genitalia or incompatibility between gonad and genital expression underwent explorative laparotomy in our institute. After laparoscopic technique was introduced in 1991, we had performed diagnostic and therapeutic laparoscopic procedures on 7 intersex children for 5 years. The laparoscopic procedure and advantage of this technique were reported historically compared with open laparotomy technique.

### PATIENTS AND METHODS

From 1985 to 1996, 11 children aged 3 months to 15 years old were referred to our institute for evaluation and management of intersex disorders (Table I). Eight children were raised as female and 3 as male. Five patients presented with ambiguous genitalia, 3 with growth retardation, each of 3 with nonpalpable testes, vaginal atresia associated with unilateral renal agenesis, and congenital adrenal hyperplasia, respectively. One (case No. 6) is a patient of Drash syndrome who had undergone radical nephrectomy for Wilm's tumor at 15 months old and has proteinuria due to glomerulopathy. Case No. 11 is a patient of lipoid adrenal hyperplasia who was born with pigmentation and general weakness. Enzymatic deficiency in the initial step of steroid synthesis pathways was elucidated by thorough endocrinological analysis.

Intraperitoneal pelvic exploration was indicated because of: (1) the presence of Y element in chromosome despite phenotypically female external genitalia (case Nos. 1–6 and 11), (2) ambiguous genitalia and suspected existence of testicular element (confirmed by human chorionic gonadotropin stimulation) despite 46,XX karyotype (case 7,8), (3) chromosomal mozaicism (46,XY/45,X) despite phenotypically male external genitalia (case 10), and (4) vaginal atresia (case 9).

The initial 4 cases (case Nos. 1–4, group 1) underwent explorative laparotomy via the lower abdominal transverse (Pfannenstiel) incision. The latter 7 cases (case Nos. 5–11, group 2) underwent laparoscopic procedure. Storz laparoscope (10 mm, 0°) was introduced via a 10 mm diameter of surgical port which was placed by open technique following about 15 mm long transverse infraumbilical incision. Pneumoperitoneum was performed using carbon dioxide with about 8 mmHg intraabdominal pressure. During the laparoscopic procedure $CO_2$ partial pressure of arterial blood was continuously monitored. If

TABLE I Patient characteristics

| Case | Age | L.S. | External genital appearance and associated abnormalities | Chromosome |
|---|---|---|---|---|
| Group 1 | | | | |
| 1 | 11 | F | Clitomegaly, labial fusion, left intralabial mass | 46XY |
| 2 | 2 | F | Clitomegaly (phallus-like), bifid scrotum | 46XYq− |
| 3 | 3M | M to F | Clitomegaly, common urogenital sinus, imperforate anus | 46XY |
| 4 | 1 | F | Clitomegaly (phallus-like), left inguinal mass | 46XY |
| Group 2 | | | | |
| 5 | 15 | F | Complete female, Turner's syndrome | 45X/46X + mar |
| 6 | 11 | F | Clitomegaly, Drash syndrome | 46XY |
| 7 | 2 | M to F | Hypospadias, bifid scrotum, nonpalpable testes | 46XX |
| 8 | 1 | F | Clitomegaly | 46XX |
| 9 | 8 | F | Vaginal atresia, vesicoureteral reflux | 46XX |
| 10 | 14 | M | Prepenile scrotum, chordee deformity | 46XY/45X |
| 11 | 1 | F | Complete female, lipoid adrenal hyperplasia | 46XY |

L.S.: legal sex.

additional laparoscopic surgery was required, 2 or 3 surgical ports were further inserted as a working port. No preoperative nasogastric tube was inserted and postoperative intraperitoneal drain was placed for 1–2 days when necessary.

### RESULTS

### Surgical Procedures in 11 Patients

Initial 4 cases in group 1 underwent explorative laparotomy. In 2 patients bilateral intraperitoneal gonadectomy was performed, while bilateral orchiectomy via local or inguinal incision was performed in other 2 patients (Table II). Urogenital endoscopy was simultaneously performed in 3 to confirm the anatomy of urogenital tract and simultaneous genitoplastic surgery was also done in 2.

In the latter 7 cases in group 2 which underwent laparoscopic exploration, 4 gonadectomies (laparoscopic procedure in 3, open inguinal approach in 1), 6 urogenital endoscopies and 2 genitoplastic surgeries were performed as one stage operative session (Table II).

Following evaluation about gonadal and ductal structures by laparotomy or laparoscopy (Table III), preferable gender was determined. Gender assignment was transformed from male to female in 2 patients (Table I). Final diagnosis was male pseudohermaphroditism in 4, mixed gonadal dysgenesis in 3, true hermaphroditism in 2, XX and XY gonadal dysgenesis in 1 each (Table III).

Surgical complications were not seen except for case No. 6 with mild fever for 3 days postoperatively.

Figure 1 is the representative laparoscopic gonadal view in the patient No. 5. She had complete normal female genitalia associated with 45X/46X + mar so that she had been followed up as a Turner's syndrome until puberty. At the age of 14, deep voice along with high serum testosterone level was noticed. The laparoscopic gonadectomy was uneventfully performed. Pathologic examination proved left streak gonad with fallopian tube and right dysplastic testis with fallopian tube and vas deference. The diagnosis was re-established as a mixed gonadal dysgenesis.

### Advantages of Laparoscopic Exploration

Mean total operation time in group 1 and 2 was 215 and 216 min respectively (Table II). Mean

TABLE II Results of operative procedure

| Case | Intraperitoneal operation | Adjunctive surgery | Total op. time (min) | Hospital stay (d) |
|---|---|---|---|---|
| Group 1 | | | | |
| 1 | — | Bilateral orchiectomy, herniorraphy, endoscopy | 125 | 10 |
| 2 | Bilateral gonadectomy | Clitovaginoplasty, endoscopy | 260 | 8 |
| 3 | — | Bilateral inguinal orchiectomy, endoscopy | 205 | 22 |
| 4 | Bilateral gonadectomy | Clitoplasty | 270 | 8 |
| Group 2 | | | | |
| 5 | Bilateral gonadectomy | — | 120 | 9 |
| 6 | Bilateral gonadectomy | Clitolabioplasty, endoscopy, needle renal biopsy | 480 | 9 |
| 7 | — | Endoscopy | 180 | 10 |
| 8 | Right gonadectomy, biopsy of left internal sexual organ | Clitoplasty, endoscopy | 400 | 6 |
| 9 | — | Endoscopy | 100 | 3 |
| 10 | — | Endoscopy, left testicular biopsy | 100 | 6 |
| 11 | — | Bilateral inguinal orchiectomy, endoscopy | 130 | 5 |
| | | mean | 215.5 | 8.7 |

—: observation alone.

TABLE III Surgical procedures and diagnoses

| Case | Gonads* | | Internal ductal derivatives | | Final diagnosis |
|---|---|---|---|---|---|
| | Right | Left | Mullerian | Wolffian | |
| Group 1 | | | | | |
| 1 | Intralabial (testis) | Intralabial (testis) | — | Epididymis, vas | XY gonadal dysgenesis |
| 2 | Abdominal (testis) | Abdominal (streak) | Hemiuterus, fallopian tube | Epididymis, vas | Mixed gonadal dysgenesis |
| 3 | Inguinal (testis) | Inguinal (testis) | — | Epididymis, vas | Male pseudohermaphroditism |
| 4 | Abdominal (streak) | Inguinal (testis) | — | Epididymis, vas | Male pseudohermaphroditism |
| Group 2 | | | | | |
| 5 | Abdominal (testis) | Abdominal (streak) | Fallopian tube, uterus | — | Mixed gonadal dysgenesis |
| 6 | Abdominal (testis) | Abdominal (streak) | Fallopian tube, uterus | — | Mixed gonadal dysgenesis |
| 7 | Abdominal (ovary**) | Abdominal (testis**) | Fallopian tube, uterus | — | True hermaphroditism |
| 8 | Abdominal (ovotestis) | Abdominal (ovary-like) | Fallopian tube, uterus | Vas | True hermaphroditism |
| 9 | Absent | Absent | — | — | XX gonadal dysgenesis |
| 10 | intrascrotal | intrascrotal | — | Epididymis, vas | Male pseudohermaphroditism |
| 11 | Inguinal | Inguinal | — | Epididymis, vas | Male pseudohermaphroditism |

* Histological findings in parentheses.
** Not biopsied.

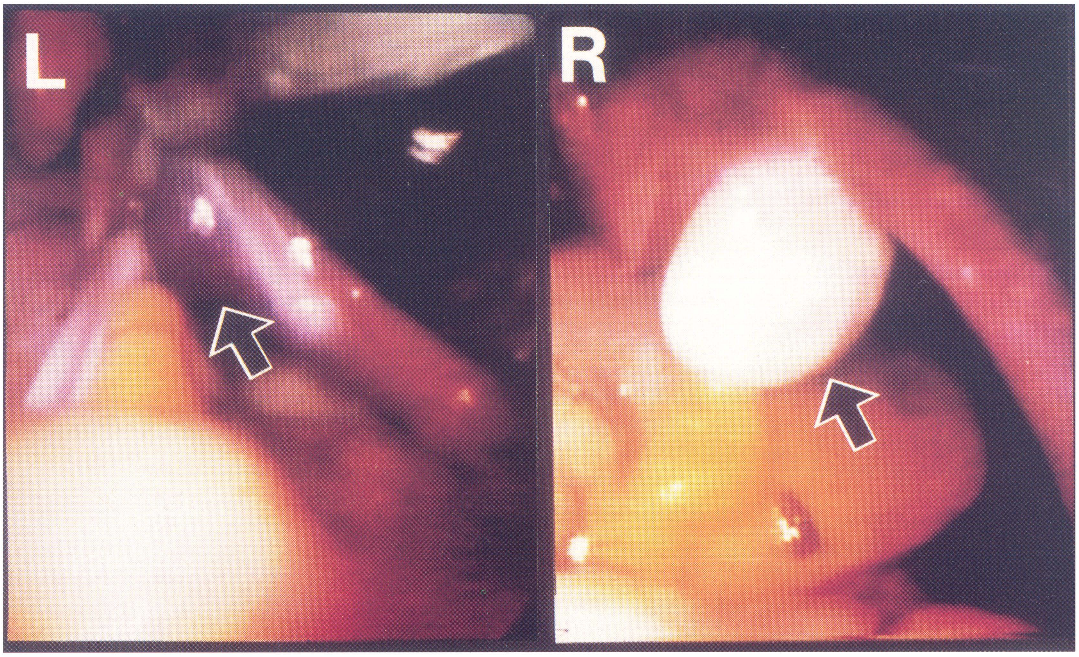

FIGURE 1 Actual photograph of laparoscopy in patient No. 5 with mixed gonadal dysgenesis. L: Left abdominal streak gonad. It had a thin, pale, structure located along the pelvic wall. R: Right abdominal testis covered with tunica albiginea.

postoperative hospital stay was shorter in group 2 than in group 1 (6.9 vs 12.0 days). However, there was no statistical difference between 2 groups (unpaired $t$ test). Postoperative analgesics were seldom used in all of the patients.

Long-term wound result of appearance in two each surgical approaches was presented in Figs. 2 and 3. Wound scar of surgical ports which had been used for laparoscopic procedure was negligible (Fig. 3). Regarding cosmetic aspects the results of laparoscopic group were superior.

### DISCUSSION

The indications of abdominal exploration in children with intersex disorders have been well presented [1]. Abdominal exploration is required in

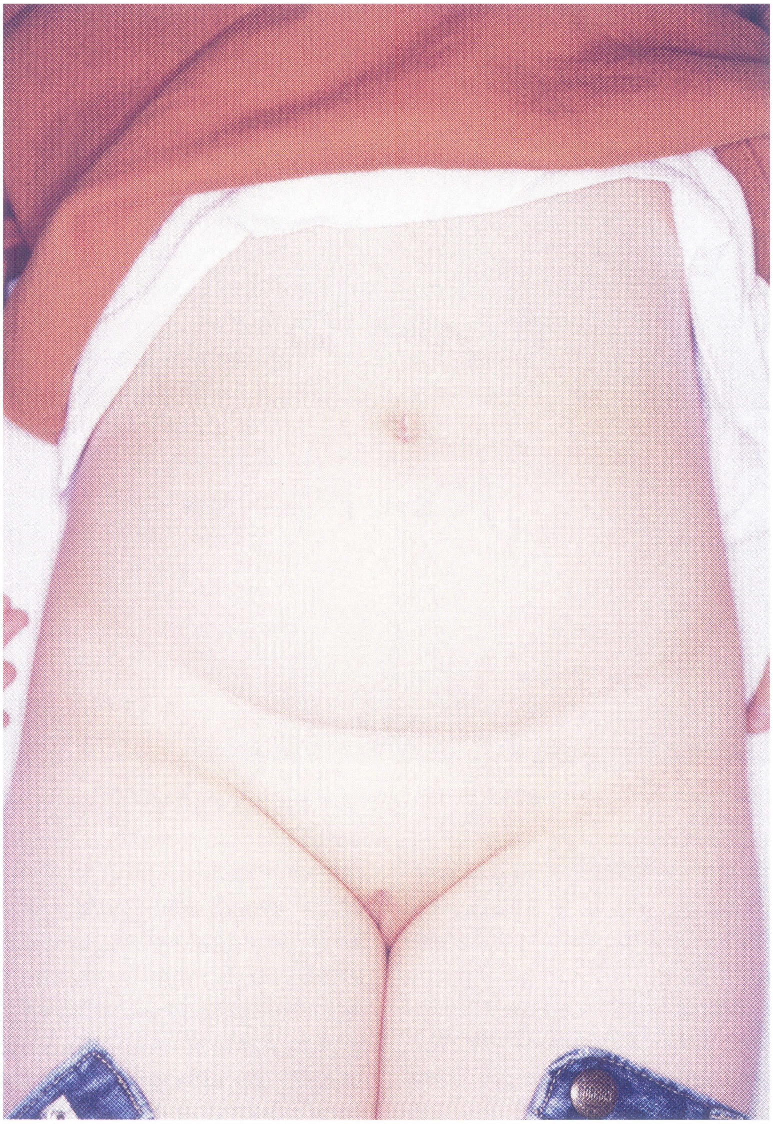

FIGURE 2 Abdominal appearance of patient No. 2. She underwent bilateral gonadectomy via the Pfannenstiel incision 9 years ago.

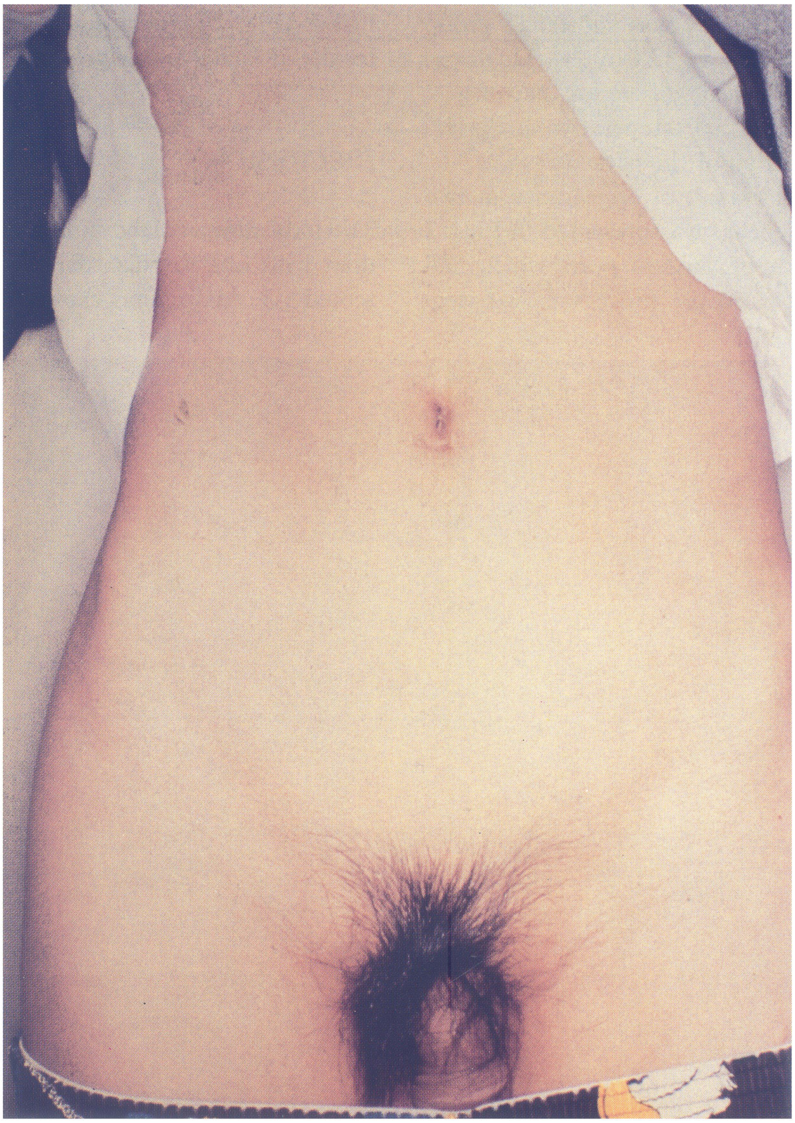

FIGURE 3 Abdominal appearance of patient No. 10. He underwent laparoscopy via the infraumbilical incision 1 year ago.

these children to establish a diagnosis and adjustable gender assignment as well as to ablate dysgenetic gonads which may have a malignant potential in the future [2,3]. The use of laparoscopic technique in intersex children is not anecdotal any more, but now widespread for the evaluation and management of these children [4,5]. Laparoscopic surgery for intersex children has two significant aspects: diagnostic and ablative purposes. Diagnostic procedure includes morphological and histological confirmation about gonads and internal ductal structures. Just after the laparoscopic evaluation, ablative treatment can be simultaneously performed such as gonadectomy, herniorrhaphy and hysterosalpingectomy along with the diagnosis and gender identity [6]. Advantage of laparoscopic procedure over explorative laparotomy is especially seen in such a case in whom ablative surgery is not necessary (Nos. 7, 9, 10 and 11 in our series).

Observational laparoscopy alone was enough in these cases.

Laparoscopic surgery in children is not completely equal to that in adults. Due to anatomical proportions in pediatric patients different technical aspects have to be considered. Elasticity of the abdominal wall and small distance to the vertebral column increase the risk of iatrogenic injuries when placing the first trocar [7]. We properly inserted the first trocar by open-laparotomy technique in all cases to avoid inadvertent injuries. As long as these miniature techniques are considered, laparoscopic exploration and combined genitoplastic surgery could resolve medical and social problems for intersex patients minimal-invasively in early childhood [8].

Laparoscopy has been obviously effective in the diagnosis of a variety of intersex spectrums. The presence and maturity of internal pelvic organs can be documented clearly through a fine-vision optical lens in magnified view and any gonadal structures can be biopsied or removed if indicated. In intersex patients diagnostic exploration would appear to be better performed laparoscopically than by an open surgical approach [4]. In our study operation time, use of analgesics, postoperative hospital stay period (which would be longer in Japan thanks to the full government coverage) and postoperative complications were not different between the two groups. However, laparoscopic inspection of internal gonad could be easily performed in shorter time with accuracy compared with open procedure. We could appropriately make the therapeutic surgical strategy including laparoscopic gonadectomy. There is no doubt that laparoscopy is a safe and reliable diagnostic tool even for intersex child.

Our data presented relatively longer hospital stay of patients than that in other countries. It could be explained by Japanese medical pay system. In Japan children with inborn abnormalities are priviledged to pay no expense for the diagnosis and management of main illness. Under these backgrounds, demands for shorter hospital stay are not of parents or ours.

Moreover, we can stress the cosmetic advantage of laparoscopic surgery in children. Most patients with ambiguous genitalia, especially those with nonpalpable gonads, were assigned as a female gender (regarding to the legal sex, 10/11 were female in this series). Indeed in these patients cosmetic outcome of the genitoplasty is one of the most important factors, while better cosmetic abdominal image should be also desirable for their social life in the future.

### CONCLUSIONS

Laparoscopic procedure is identical with open exploration for diagnosis and treatment in intersex patients but provides a more acceptable cosmetic result. This technique should become a standard diagnostic and therapeutic method in pediatric urology.

### *References*

[1] Donahoe, P.K. Diagnosis and management of newborns and infants with ambiguous genitalia. In: Ashcraft, K.W. *Pediatric Urology* First Edition. W. B. Saunders Company, Philadelphia, 1990.

[2] Manuel, M., Katayama, K.P., Jones, H.W. *et al.* The age of occurrence of gonadal tumors in inter sex patient with a Y chromosome. *Am. J. Obstet. Gynec.* 1976; **124**: 293–300.

[3] Borer, J.G., Nitti, V.W. and Glassberg, K.I. Mixed gonadal dysgenesis and dysgenetic male pseudohermaphroditism. *J. Urol.* 1995; **153**: 1267–1273.

[4] Clayman, R.V. Pediatric laparoscopy: Quo vadis? A view from the outside. *J. Urol.* 1994; **152**: 730–733.

[5] Duckett, J.W. Editorial: Pediatric laparoscopy: Prudence, please. *J. Urol.* 1994; **151**: 742–743.

[6] Yu, T.J., Shu, K., Kung, F.T., *et al.* Use of laparoscopy in intersex patients. *J. Urol.* 1995; **154**: 1193–1196.

[7] Seibold, J., Janetschek, G. and Bartsch, G. Laparoscopic surgery in pediatric urology. *Eur. Urol.* 1996; **30**: 394–399.

[8] Fukuzaki, A., Chiba, Y. and Orikasa, S. Diagnostic laparoscopy in urology. *Jpn. J. Endourol. ESWL* 1994; **7**: 23–25.

